# Regulation of Osteoblast Differentiation by Acid-Etched and/or Grit-Blasted Titanium Substrate Topography Is Enhanced by 1,25(OH)_2_D_3_ in a Sex-Dependent Manner

**DOI:** 10.1155/2015/365014

**Published:** 2015-04-06

**Authors:** Rene Olivares-Navarrete, Sharon L. Hyzy, Barbara D. Boyan, Zvi Schwartz

**Affiliations:** ^1^Department of Biomedical Engineering, School of Engineering, Virginia Commonwealth University, 601 W. Main Street, Richmond, VA 23284, USA; ^2^Department of Biomedical Engineering, Georgia Institute of Technology, 313 Ferst Drive NW, Atlanta, GA 30332, USA; ^3^Department of Periodontics, University of Texas Health Science Center at San Antonio, 7703 Floyd Curl Drive, San Antonio, TX 78229, USA

## Abstract

This study assessed contributions of micron-scale topography on clinically relevant titanium (Ti) to differentiation of osteoprogenitor cells and osteoblasts; the interaction of this effect with 1*α*,25-dihydroxyvitamin D_3_ (1*α*,25(OH)_2_D_3_); and if the effects are sex-dependent. Male and female rat bone marrow cells (BMCs) were cultured on acid-etched (A, *R*_*a*_ = 0.87 *μ*m), grit-blasted (GB, *R*_*a*_ = 3.90 *μ*m), or grit-blasted/acid-etched (SLA, *R*_*a*_ = 3.22 *μ*m) Ti. BMCs were sensitive to surface topography and underwent osteoblast differentiation. This was greatest on SLA; acid etching and grit blasting contributed additively. Primary osteoblasts were also sensitive to SLA, with less effect from individual structural components, demonstrated by enhanced local factor production. Sex-dependent responses of BMCs to topography varied with parameter whereas male and female osteoblasts responded similarly to surface treatment. 1*α*,25(OH)_2_D_3_ enhanced cell responses on all surfaces similarly. Effects were sex-dependent and male cells grown on a complex microstructured surface were much more sensitive than female cells. These results indicate that effects of the complex SLA topography are greater than acid etching or grit blasting alone on multipotent BMCs and committed osteoblasts and that individual parameters are sex-specific. The effect of 1*α*,25(OH)_2_D_3_ was sex dependent. The results also suggest that levels of 1*α*,25(OH)_2_D_3_ in the patient may be important in osseointegration.

## 1. Introduction

Current dental practice employs implants with a variety of surface modifications, yielding improved bone-to-implant contact and patient outcomes. Alterations in surface microtopography change the adsorption of proteins to the implant surface, which also affects cell attachment and differentiation [1–3]. Many studies, including those from our group, have shown that surface microroughness influences osteoblast response [4–6]. A series of studies assessing the role of specific surface properties using electro-micromachined, acid-etched, or grit-blasted/acid-etched titanium (Ti) substrates showed that the greatest osteoblast differentiation was present on the more topographically complex surfaces, with both micron- and submicron-scale features [7–9].

For an implant to become osseointegrated, cells that migrate to the area must attach to the surface and then differentiate into mature osteoblasts. Recently, we demonstrated that commercially available human mesenchymal stem cells are also sensitive to Ti surface microtopography and exhibit osteoblast differentiation even in the absence of media supplements typically used to promote mineralized bone nodule formation [6]. Wnt5a mediated the effects of the surface through the noncanonical Wnt signaling pathway [10]. Stangl et al. [11] showed that a human fetal osteoblast cell line responded preferentially to changes in microtopography of commercially pure Ti surfaces, indicating that progenitor cells in the osteoblast lineage are affected as well.

Grit blasting and acid etching are widely used in combination to modify titanium implants. Grit blasting imparts macron- and micron-scale topographic structures on implant surfaces, while acid etching creates micron-, submicron-, and nanoscale topographies. The application of these two techniques in combination creates implant surfaces with a complex topography that has been well studied in osseointegration* in vivo* [12, 13] and osteoblasts* in vitro* [14]. These studies demonstrate that the topographical features of Ti surfaces affect differentiation of osteoprogenitor cells and maturation of osteoblast lineage cells. However, less is known about the individual contributions of these substrate features to directing osteoblastic differentiation of progenitor cells or maturation of committed osteoblasts.

Several reports have shown surface-dependent differences of osteoblasts in response to osteotropic hormones such as 1*α*,25-dihydroxyvitamin D_3_ (1*α*,25(OH)_2_D_3_) [15–18]. Interestingly, not only are responses to 1*α*,25(OH)_2_D_3_ on complex microstructured surfaces greater than on smooth surfaces, but there are sex-specific differences in hormone responses as well. Our group has demonstrated that calvarial osteoblasts from male donors exhibit a more robust response to 1*α*,25(OH)_2_D_3_ than cells from female donors, increasing important osteogenic markers as well as soluble factors that increase the angiogenic and osteogenic microenvironment [15]. Similarly, sex-specific responses to a variety of stimuli have been observed in myotubes [19], angiogenesis [20], spleen, and thymus [21, 22]. These observations suggest that osteoblast cells may also respond to surface roughness modifications in a sex-dependent manner.

The aim of the present study was to evaluate the role of topographic surface features in osteogenic differentiation of rat bone marrow stromal cells (BMCs) and in the maturation of rat calvarial osteoblasts and to assess whether the effects of specific surface treatments, either alone or in combination, are sex-dependent. In addition, we examine how treatment with 1*α*,25(OH)_2_D_3_ modifies the responses of male and female primary cells to these surface topographies.

## 2. Materials and Methods

### 2.1. Preparation and Characterization of Ti Disks

Titanium (Ti) disks were prepared from 1 mm thick sheets of grade 2 unalloyed Ti (ASTM F67 “Unalloyed Titanium for Surgical Implant Applications”) with a 15 mm diameter to fit in a 24-well culture plate as previously described [16, 23, 24]. Briefly, disks were washed in acetone and processed through a 2% ammonium fluoride, 2% hydrofluoric acid, and 10% nitric acid solution at 55°C for 30 s to pretreat Ti disks. Submicron-scale rough (A) surfaces were produced by treating pretreated disks with heated, concentrated acid, resulting in *R*_*a*_ of 870 nm. GB surfaces were produced by coarse grit blasting with 0.25–0.50 mm corundum grit at 5 bars until the surface reached a uniform gray tone pretreatment disks (*R*_*a*_ = 3.90 *μ*m). To produce disks with a mixed topography (SLA), grit-blasted disks were acid-etched (*R*_*a*_ = 3.22 *μ*m). Scanning electron microscopic images and surface characterization have been described previously [8, 14].

### 2.2. Bone Marrow Cell Isolation and Response

Bone marrow cells (BMCs) were isolated from the tibias and femurs of 100–125 gram male and female Sprague-Dawley rats under Georgia Institute of Technology Institutional Animal Care and Use Committee Protocols and following appropriate guidelines. For each sex, marrow was isolated and pooled from the tibias and femurs of four animals. Marrow was flushed from the intramedullary canal of each bone into a sterile conical tube using a 5 mL syringe and 18-gauge needle. Marrow was briefly incubated with Collagenase IA (Sigma Aldrich, St. Louis, MO) to release the cells from the matrix. The cells were pelleted and plated in a flask for expansion. Cells were cultured in Mesenchymal Stem Cell Growth Media (Lonza Biosciences, Walkersville, MD). At first passage, cells were plated on tissue culture polystyrene (TCPS) or Ti surfaces (A, GB, SLA) at 5,000 cells/cm^2^ (based on a 15 mm diameter smooth surface) and grown to confluence on TCPS, typically after 7 days. At confluence, media were changed and cells incubated for an additional 24 hours.

### 2.3. Rat Osteoblast Cultures

Osteoblasts were isolated from frontal and parietal (calvaria) bones of 100–125 gram male and female Sprague-Dawley rats using enzymatic isolation as described previously [25]. Briefly, rat bones cleaned of periosteum and soft tissues were cut into 1-2 mm^2^ pieces. The bone chips were washed three times in Hank's balanced salt solution (HBSS, Invitrogen, Carlsbad, CA) containing 3% penicillin-streptomycin (Invitrogen). After washing, bone chips were digested with an enzymatic cocktail of collagenase IA and dispase (Invitrogen) in HBSS for 1 hour at 37°C. The supernatants of the first two digestions were discarded to avoid contamination by fibroblasts. The bone chips were digested three more times using the same method; at each step, the digestion media were collected and quenched with Dulbecco's modification of Eagle's medium (DMEM, cellgro, Manassas, VA) supplemented with 10% fetal bovine serum (Thermo Fisher HyClone, Waltham, MA) and 1% penicillin-streptomycin (Invitrogen). Calvaria from eight rats per sex were pooled for each experiment.

To confirm osteoblastic phenotype of isolated rat calvarial cells, we also examined cell responses to 24-hour treatment with the osteotropic hormone 1*α*,25(OH)_2_D_3_ (Enzo Life Sciences, Plymouth Meeting, PA) after confluence on TCPS. Both male and female rat cells exhibited dose-dependent decreases in cell number and increased alkaline phosphatase specific activity and osteocalcin levels in response to treatment with 10^−10 ^M, 10^−9 ^M, and 10^−8 ^M 1*α*,25(OH)_2_D_3_ (data not shown).

Validated rat osteoblasts were plated on TCPS or Ti surfaces at a density of 10,000 cells per cm^2^. Media were exchanged at 24 hours and then every 48 hours until the cells reached confluence on TCPS. At confluence, the cells were treated with vehicle (0.001% ethanol) or 10^−8 ^M 1*α*,25(OH)_2_D_3_ for 24 hours and harvested as described below.

### 2.4. Biochemical and Immunoassays

Cell number was determined in all cultures 24 hours after cells on TCPS reached confluence. Cells were released from the surfaces using two sequential 10 m incubations in 0.25% trypsin at 37°C, to ensure that any remaining cells were removed from rough Ti surfaces, and counted using an automatic cell counter (Z1 Particle Counter, Beckman Coulter, Fullerton, CA). Osteoblast differentiation and maturation were evaluated using alkaline phosphatase specific activity as an early marker and osteocalcin secretion as a later marker [26]. Cellular alkaline phosphatase specific activity (orthophosphoric monoester phosphohydrolase, alkaline; E.C. 3.1.3.1) of the cell lysates was assayed by measuring the release of *p*-nitrophenol from *p*-nitrophenylphosphate at pH 10.2 and results were normalized to total protein content of the cell lysates (Pierce BCA Protein Assay, Thermo Fisher, Rockford, IL). Levels of osteocalcin in the conditioned media were measured by immunoassay (Osteocalcin EIA, Biomedical Technologies, Stoughton, MA).

The conditioned media were also assayed for growth factors and cytokines. Active TGF-*β*1 was measured prior to acidification of the conditioned media, using an enzyme-linked immunosorbent assay (R&D Systems, Minneapolis, MN). Total TGF-*β*1 was measured after acidifying the media and latent TGF-*β*1 was defined as total TGF-*β*1 minus active TGF-*β*1. Osteoprotegerin (OPG) was measured using an ELISA kit (DuoSet, R&D Systems, Minneapolis, MN). Vascular endothelial growth factor (VEGF) was measured in the conditioned media of BMCs using an ELISA kit (R&D Systems). Immunoassays were normalized to total cell number.

### 2.5. Statistical Analysis

Data presented are treatment/control ratios from one of two experiments, both with comparable results. Responses on TCPS serve as controls. For each experiment, each variable was tested in six independent cultures. Data were first analyzed by ANOVA; when statistical differences were detected, a post hoc analysis of Bonferroni's modification of Student's *t*-test was used. *P* values <0.05 were considered to be significant.

## 3. Results

**Figure 1:**
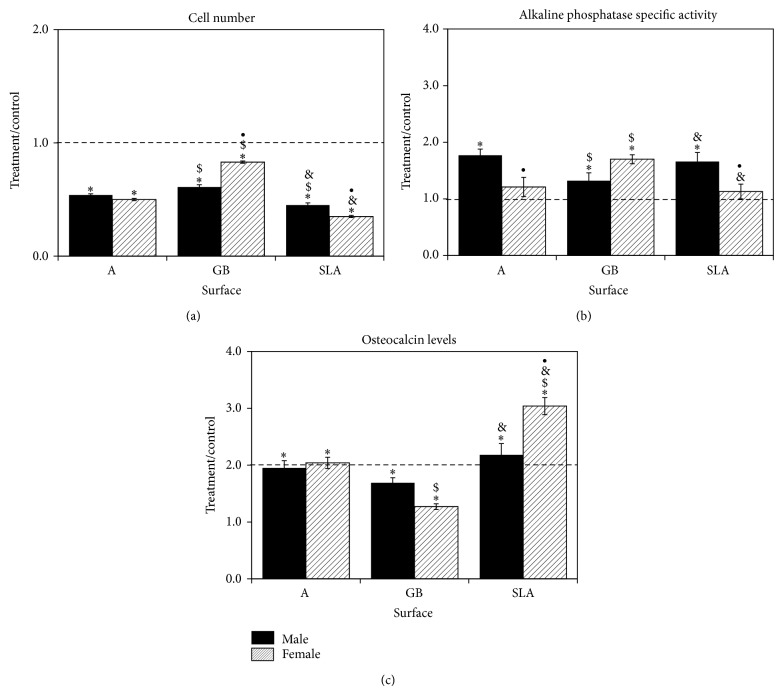
Response of rat BMCs to microstructured titanium surfaces. Male and female BMCs were cultured on TCPS, A, GB, or SLA surfaces and grown to confluence. Cell number (a), alkaline phosphatase specific activity (b), and osteocalcin levels (c) were measured. Data are displayed as treatment/control of cells on Ti surfaces to cells on TCPS. ^*^ *P* < 0.05 versus TCPS; ^$^ *P* < 0.05 versus A surface; ^&^ *P* < 0.05 versus GB; ^•^ *P* < 0.05, female versus male.

BMCs had lower cell number when cultured on Ti substrates in comparison to TCPS (Figure 1(a)). This effect was significantly lower on SLA compared to GB and A and significantly lower on A than GB. Female cells had higher cell number on GB and lower cell number on SLA than male cells. Alkaline phosphatase specific activity was sensitive to surface topography in a sex-specific manner (Figure 1(b)). Activity was increased in male cells on A and SLA in comparison to TCPS. However, in female BMC cultures, activity was increased only on in cells on GB. Both male and female BMCs exhibited increased osteocalcin in their conditioned media on all surfaces (SLA > A > GB) (Figure 1(c)). This effect was comparable in male and female cultures on GB surfaces, but cells isolated from female rats produced more osteocalcin on SLA than males.

**Figure 2:**
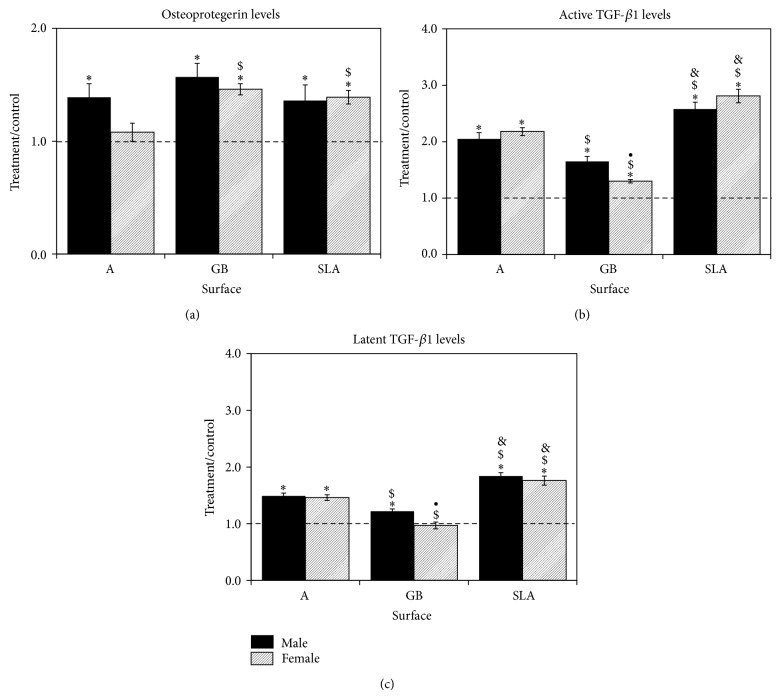
Response of rat BMCs to microstructured titanium surfaces. Male and female BMCs were cultured on TCPS or Ti disks and grown to confluence. OPG (a), active TGF-*β*1 (b), and latent TGF-*β*1 (c) were measured in the conditioned media. Data are displayed as treatment/control of cells on Ti surfaces to cells on TCPS. ^*^ *P* < 0.05 versus TCPS; ^$^ *P* < 0.05 versus A surface; ^&^ *P* < 0.05 versus GB; ^•^ *P* < 0.05, female versus male.

OPG was increased in male cells by 30–50% on all surfaces (Figure 2(a)). Female BMCs showed a comparable increase in OPG on GB and SLA surfaces over levels on TCPS when compared to males. However, female cells cultured on A surfaces did not exhibit increased OPG in comparison to TCPS. Male BMCs produced more active (Figure 2(b)) and latent (Figure 2(c)) TGF-*β*1 on all Ti substrates (SLA > A > GB) in comparison to TCPS. Female cells produced similar levels of active TGF-*β*1 on A and SLA in comparison to male cells; however, they secreted less active and latent TGF-*β*1 on GB than male cells.

**Figure 3:**
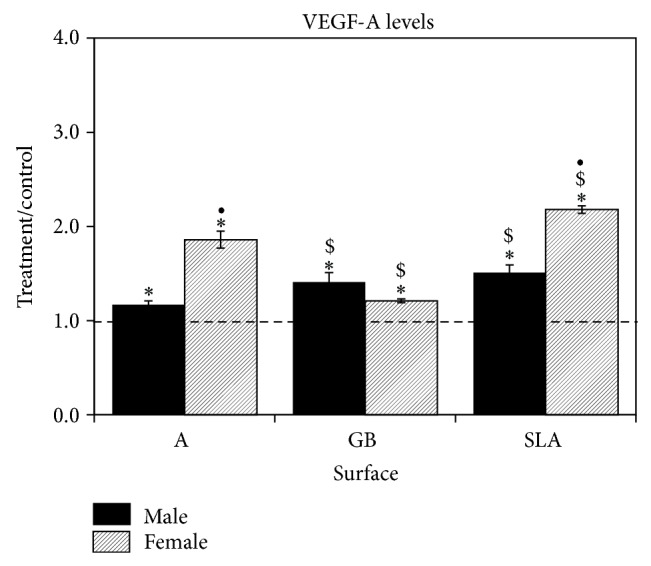
Response of rat BMCs to microstructured titanium surfaces. Male and female BMCs were cultured on TCPS or Ti disks and grown to confluence. VEGF was measured in the conditioned media. Data are displayed as treatment/control of cells on Ti surfaces to cells on TCPS. ^*^ *P* < 0.05 versus TCPS; ^$^ *P* < 0.05 versus A surface; ^&^ *P* < 0.05 versus GB; ^•^ *P* < 0.05, female versus male.

VEGF was also produced in a surface-dependent, sex-specific manner by BMC cells (Figure 3). Male cells produced more VEGF on GB and SLA than on TCPS. In contrast, female cells produced more VEGF on A and SLA than on GB. Female cells produced more VEGF-A on acid-etched A and SLA surfaces than male cells.

**Figure 4:**
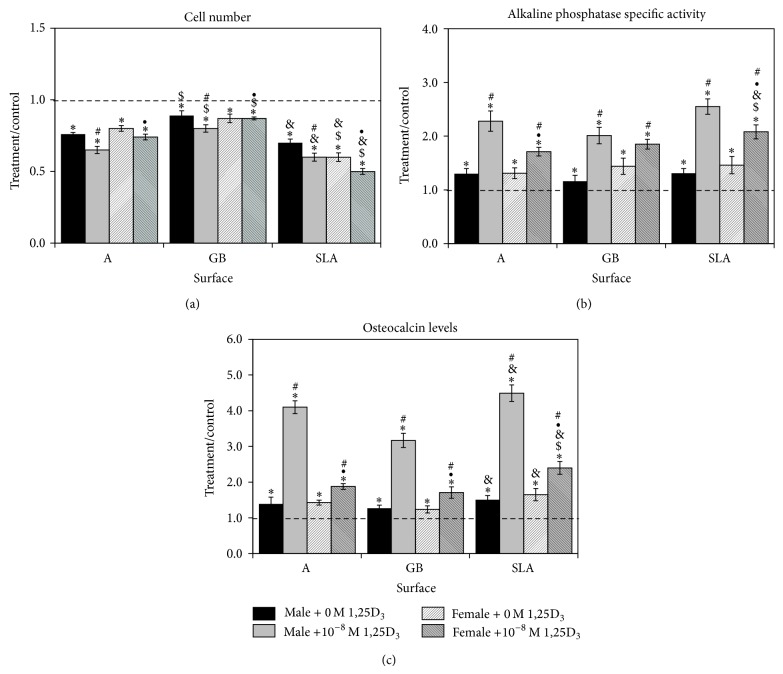
Effect of Ti surface topography with or without 1*α*,25(OH)_2_D_3_ on osteoblast differentiation of rat calvarial osteoblasts. Male and female calvarial osteoblasts were cultured on TCPS or Ti disks. At confluence, cells were treated for 24 hours with 10^−8 ^M 1*α*,25(OH)_2_D_3_. Cell number (a), alkaline phosphatase specific activity (b), and osteocalcin levels (c) were measured. Data are displayed as treatment/control of cells on Ti surfaces to cells on TCPS. ^*^ *P* < 0.05 versus TCPS; ^$^ *P* < 0.05 versus A surface; ^&^ *P* < 0.05 versus GB; ^•^ *P* < 0.05, female versus male; ^#^ *P* < 0.05  versus 0 M 1*α*,25(OH)_2_D_3_.

Osteoblasts had reduced cell numbers when cultured on Ti surfaces when compared to TCPS (Figure 4(a)). This was less pronounced in cultures grown on GB surfaces compared to A and SLA surfaces and was most pronounced on SLA. 1*α*,25(OH)_2_D_3_ enhanced the decreased cell number of cells grown on SLA surface. In male cells on all surfaces decreased cell number but only affected female cells on SLA. Alkaline phosphatase specific activity was greater in osteoblast cultures grown on Ti substrates in comparison to TCPS (Figure 4(b)). 1*α*,25(OH)_2_D_3_ increased activity on all surfaces examined. Alkaline phosphatase specific activity was significantly stimulated in osteoblasts from male rats in comparison to female cells. Levels of osteocalcin in the conditioned media were higher in osteoblast cultures on all Ti surfaces compared to TCPS (Figure 4(c)). 1*α*,25(OH)_2_D_3_ increased osteocalcin in male and female osteoblast cultures, but the stimulatory effect was significant and more than three times greater in the male cells compared to female cells. Moreover, the stimulatory effects of 1*α*,25(OH)_2_D_3_ were less robust on GB than on A or SLA in the male cells.

**Figure 5:**
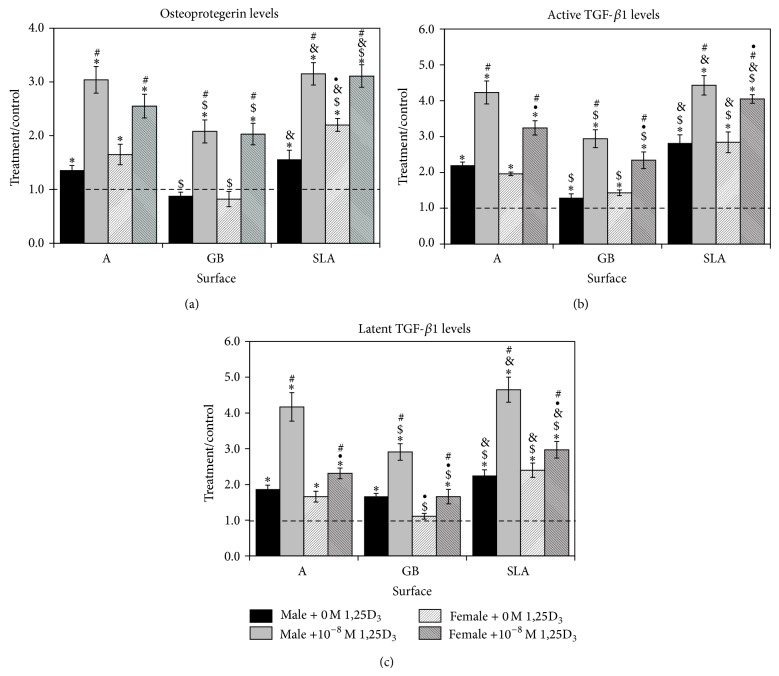
Effect of Ti surface topography with or without 1*α*,25(OH)_2_D_3_ on osteoblast differentiation of rat calvarial osteoblasts. Male and female calvarial osteoblasts were cultured on TCPS or Ti disks. At confluence, cells were treated for 24 hours with 10^−8 ^M 1*α*,25(OH)_2_D_3_. OPG (a), active TGF-*β*1 (b), and latent TGF-*β*1 (c) were measured in the conditioned media. Data are displayed as treatment/control of cells on Ti surfaces to cells on TCPS. ^*^ *P* < 0.05 versus TCPS; ^$^ *P* < 0.05 versus A surface; ^&^ *P* < 0.05 versus GB; ^•^ *P* < 0.05, female versus male; ^#^ *P* < 0.05 versus 0 M 1*α*,25(OH)_2_D_3_.

OPG was also increased in osteoblast cultures on Ti surfaces in comparison to TCPS (SLA > A > GB) (Figure 5(a)). This was even more evident in female osteoblast cultures. 1*α*,25(OH)_2_D_3_ increased OPG by more than 100% in osteoblasts on all surfaces (A, SLA > GB) with no difference between male and female. The response of female osteoblasts was less robust on A and SLA but was comparable to the male cells on GB. Active TGF-*β*1 was increased in male and female osteoblasts on all surfaces in comparison to control (SLA < A < GB) (Figure 5(b)). Treatment with 1*α*,25(OH)_2_D_3_ caused significant increases in TGF-*β*1 in male and female cells on all surfaces (GB < A < SLA). In the untreated osteoblasts, there was no difference between male and female cells in TGF-*β*1 levels. However, treatment with 1*α*,25(OH)_2_D_3_ caused a statistically greater increase in male cells than in female cells on A and GB surfaces; however, there was no difference on SLA substrates. Latent TGF-*β*1 was increased in male osteoblasts on all surfaces in comparison to control and in female osteoblasts grown on A and SLA surfaces; female osteoblasts on GB surfaces were not significantly different from control (Figure 5(c)). Treatment with 1*α*,25(OH)_2_D_3_ increased latent TGF-*β*1 in male and female cells on all surfaces following a similar pattern (GB < A < SLA). Male cells had significantly higher levels of latent TGF-*β*1 than females on all surfaces after treatment with 1*α*,25(OH)_2_D_3_; untreated female osteoblasts had lower levels of TGF-*β*1 on GB surfaces than male cells.

## 4. Discussion

This study examined the effects of different clinically relevant surfaces on osteoprogenitor differentiation and osteoblast maturation. BMCs grown on Ti surfaces that were acid-etched, grit-blasted, or grit-blasted and acid-etched exhibited a similar reduction in cell number and enhancement of alkaline phosphatase activity and OPG levels. However, the SLA surface, which is the combination of acid etching and grit blasting, significantly enhanced in an additive manner the production of osteocalcin, active and latent TGF-*β*1, and VEGF in comparison to the other surfaces. These results using primary rat BMCs were similar to the effects of SLA on human mesenchymal stem cells obtained from Lonza that were described previously [6]. The results also demonstrated that the effect of the surface was sex-dependent for some but not all parameters. The clinical importance of this observation in females needs to be examined.

Osteoblast cultures grown on the same Ti surfaces showed similar results with respect to proliferation and maturation as BMCs: reduced proliferation and enhanced production of osteocalcin, OPG, and active and latent TGF-*β*1. The effects of surface topography were enhanced on SLA, as has been noted previously for the human osteoblast-like MG63 cell line [5, 16, 27]. Both male and female osteoblasts respond similarly to the different surfaces, with the exception of increased levels of OPG in cultures of female cells grown on SLA. These results may indicate that both sexes can osseointegrate well with the appropriate surface and that SLA has a better ability to enhance osteoblastic differentiation and maturation in comparison to either processing method alone.

Establishment of a healthy vasculature is critical for implant osseointegration [28]. Both male and female cells increased levels of VEGF in response to Ti surfaces, indicating the cells have begun to signal for new vasculature in response to the surface. However, while male BMCs showed a greater increase in VEGF on GB and SLA surfaces, female BMCs increased VEGF production in response to the submicron-scale topographic features induced by acid etching. Similar studies using human alveolar osteoblasts have shown that VEGF production is increased in cells grown on A and SLA surfaces [29], and previous studies from our group showed that VEGF levels also increased in human mesenchymal stem cells and MG63 cells in response to surface topography [6, 30]. Additionally, in vitro studies have shown that androgen induced a sex-dependent effect on angiogenesis in a Matrigel assay [20].

Previously we demonstrated that fetal rat calvarial osteoblasts are more sensitive to treatment with 1*α*,25(OH)_2_D_3_ when cultured on Ti substrates [31]. In the present study, we sought to decouple the topographic features that enhanced osteoblast response to 1*α*,25(OH)_2_D_3_ using adult rat osteoblasts. The rat osteoblasts cultured on Ti respond to 1*α*,25(OH)_2_D_3_ with decreased cell number and increased alkaline phosphatase specific activity and osteocalcin production in comparison to cells cultured on TCPS. In contrast, this differential response to the vitamin D metabolite may be due to an increased maturation state of the cells grown on Ti substrates at the time of treatment, as has been demonstrated in other studies [31–33].

The effects of 1*α*,25(OH)_2_D_3_ on the rat osteoblasts were sex-dependent. Treatment with 1*α*,25(OH)_2_D_3_ increased OPG protein levels in both male and female osteoblasts, although this effect was greater in males. This confirms previous results in our laboratory demonstrating upregulation of OPG mRNA expression and protein levels after treatment with 1*α*,25(OH)_2_D_3_ in human osteoblast and MG63 cells cultured on SLA surfaces [15, 34]. Interestingly, treatment of male osteoblasts with 1*α*,25(OH)_2_D_3_ increased latent TGF-*β*1 more than active TGF-*β*1; in female osteoblasts, the greatest response was in active TGF-*β*1. Previous studies indicate that active TGF-*β*1 increases in response to 1*α*,25(OH)_2_D_3_ treatment, possibly due to activation of latent TGF-*β*1 [35, 36]. However, the dimorphism in the response suggests that in female osteoblasts 1*α*,25(OH)_2_D_3_ shifts TGF-*β*1 levels towards the active form to inhibit bone remodeling [37], while in male cells there is more latent TGF-*β*1 to allow cells to progress towards terminal osteoblast differentiation [32].

1*α*,25(OH)_2_D_3_ enhanced osteoblast maturation and inhibited osteoblast proliferation on all surfaces. When calculated as fold increase, this effect was similar on all surfaces examined. These results indicate that each of these surfaces affects the osteoblast phenotype and makes cells more sensitive to the hormone effect.

The effect of 1*α*,25(OH)_2_D_3_ on osteoblast cells grown on rough surfaces was sex-dependent since the enhancement of all the parameters examined was significantly higher in male cells in comparison to female cells. These results are in agreement with other studies that have found that femoral neck osteoblasts from males have larger increases in osteocalcin secretion in response to 1*α*,25(OH)_2_D_3_ than female cells [38]. Clinically, vitamin D deficiency has been correlated with decreased bone mineral density in male, but not female, patients [39]. These results may indicate that males are more sensitive to vitamin D levels and suggest that if we would like to achieve maximum osseointegration clinically, especially in compromised cases, we could examine the patient's vitamin D levels and supplement as needed if levels are outside the normal range.

The results of the present study indicate that BMCs, the first cells to recognize the surface* in vivo*, are sensitive to surface topography and can undergo osteogenic differentiation in response to surface cues. The effect was mainly in response to the complex SLA topography and the surface treatments (acid etching and grit blasting) contribute additively to this effect. BMC response to the topographies in this study exhibited sex dependence in some parameters, indicating that they are differentially regulated by both substrate structural elements and cell sex. Primary osteoblasts were also sensitive mainly to the complex SLA topography, with less effect of the individual structural components. Osteoblast response to topography was not sex-dependent to the extent seen in the BMCs. Treatment with 1*α*,25(OH)_2_D_3_ enhanced osteoblast response to the surfaces examined, with a similar fold increase on all surfaces. The response of osteoblasts to 1*α*,25(OH)_2_D_3_ is sex-dependent and male cells grown on a complex microstructure surface are much more sensitive 1*α*,25(OH)_2_D_3_ treatment.

## 5. Conclusions

These results demonstrate that both surface roughness and systemic hormones can affect bone formation at the implant site. While males and females have similar responses to surface roughness, they differ in production of local factors regulating bone resorption and in the magnitude of the response 1*α*,25(OH)_2_D_3_. These factors are important as future modifications are made to tailor implants to sex-specific differences to improve osseointegration and long-term implant life.

## References

[1] Cai K., Bossert J., Jandt K. D. (2006). Does the nanometre scale topography of titanium influence protein adsorption and cell proliferation?. *Colloids and Surfaces B: Biointerfaces*.

[2] Protivínský J., Appleford M., Strnad J., Helebrant A., Ong J. L. (2007). Effect of chemically modified titanium surfaces on protein adsorption and osteoblast precursor cell behavior. *International Journal of Oral and Maxillofacial Implants*.

[3] Hao L., Lawrence J. (2007). Wettability modification and the subsequent manipulation of protein adsorption on a Ti6Al4V alloy by means of CO_2_ laser surface treatment. *Journal of Materials Science: Materials in Medicine*.

[4] Brunette D. M., Ratkay J., Chehroudi B. (1991). *Behaviour of Osteoblasts on Micromachined Surfaces*.

[5] Martin J. Y., Schwartz Z., Hummert T. W. (1995). Effect of titanium surface roughness on proliferation, differentiation, and protein synthesis of human osteoblast-like cells (MG63). *Journal of Biomedical Materials Research*.

[6] Olivares-Navarrete R., Hyzy S. L., Hutton D. L. (2010). Direct and indirect effects of microstructured titanium substrates on the induction of mesenchymal stem cell differentiation towards the osteoblast lineage. *Biomaterials*.

[7] Boyan B. D., Lossdörfer S., Wang L. (2003). Osteoblasts generate an osteogenic microenvironment when grown on surfaces with rough microtopographies. *European Cells and Materials*.

[8] Zhao G., Zinger O., Schwartz Z., Wieland M., Landolt D., Boyan B. D. (2006). Osteoblast-like cells are sensitive to submicron-scale surface structure. *Clinical Oral Implants Research*.

[9] Zinger O., Zhao G., Schwartz Z. (2005). Differential regulation of osteoblasts by substrate microstructural features. *Biomaterials*.

[10] Olivares-Navarrete R., Hyzy S. L., Hutton D. L. (2011). Role of non-canonical Wnt signaling in osteoblast maturation on microstructured titanium surfaces. *Acta Biomaterialia*.

[11] Stangl R., Rinne B., Kastl S. (2001). The influence of pore geometry in CP Ti-implants—a cell culture investigation. *European Cells and Materials*.

[12] Cochran D. L., Nummikoski P. V., Higginbottom F. L., Hermann J. S., Makins S. R., Buser D. (1996). Evaluation of an endosseous titanium implant with a sandblasted and acid-etched surface in the canine mandible: radiographic results. *Clinical Oral Implants Research*.

[13] Bornstein M. M., Harnisch H., Lussi A., Buser D. (2007). Clinical performance of wide-body implants with a sandblasted and acid-etched (SLA) surface: results of a 3-year follow-up study in a referral clinic. *International Journal of Oral and Maxillofacial Implants*.

[14] Zhao G., Raines A. L., Wieland M., Schwartz Z., Boyan B. D. (2007). Requirement for both micron- and submicron scale structure for synergistic responses of osteoblasts to substrate surface energy and topography. *Biomaterials*.

[15] Olivares-Navarrete R., Hyzy S. L., Chaudhri R. A., Zhao G., Boyan B. D., Schwartz Z. (2010). Sex dependent regulation of osteoblast response to implant surface properties by systemic hormones. *Biology of Sex Differences*.

[16] Boyan B. D., Batzer R., Kieswetter K. (1998). Titanium surface roughness alters responsiveness of MG63 osteoblast-like cells to 1*α*,25-(OH)_2_D_3_. *Journal of Biomedical Materials Research*.

[17] Lohmann C. H., Tandy E. M., Sylvia V. L. (2002). Response of normal female human osteoblasts (NHOst) to 17*β*-estradiol is modulated by implant surface morphology. *Journal of Biomedical Materials Research*.

[18] Schwartz Z., Bell B. F., Wang L., Zhao G., Olivares-Navarrete R., Boyan B. D. (2007). Beta-1 integrins mediate substrate dependent effects of 1*α*,25(OH)_2_D_3_ on osteoblasts. *The Journal of Steroid Biochemistry and Molecular Biology*.

[19] Salehzadeh F., Rune A., Osler M., Al-Khalili L. (2011). Testosterone or 17beta-estradiol exposure reveals sex-specific effects on glucose and lipid metabolism in human myotubes. *Journal of Endocrinology*.

[20] Sieveking D. P., Lim P., Chow R. W. Y. (2010). A sex-specific role for androgens in angiogenesis. *Journal of Experimental Medicine*.

[21] Thangavel C., Dhir R. N., Volgin D. V., Shapiro B. H. (2007). Sex-dependent expression of CYP2C11 in spleen, thymus and bone marrow regulated by growth hormone. *Biochemical Pharmacology*.

[22] Almaden Y., Felsenfeld A. J., Rodriguez M. (2003). Proliferation in hyperplastic human and normal rat parathyroid glands: role of phosphate, calcitriol, and gender. *Kidney International*.

[23] Zhao G., Schwartz Z., Wieland M. (2005). High surface energy enhances cell response to titanium substrate microstructure. *Journal of Biomedical Materials Research—Part A*.

[24] Rupp F., Scheideler L., Olshanska N., de Wild M., Wieland M., Geis-Gerstorfer J. (2006). Enhancing surface free energy and hydrophilicity through chemical modification of microstructured titanium implant surfaces. *Journal of Biomedical Materials Research A*.

[25] Bellows C. G., Aubin J. E., Heersche H. N. M., Antosz M. E. (1986). Mineralized bone nodules formed in vitro from enzymatically released rat calvaria cell populations. *Calcified Tissue International*.

[26] Lian J. B., Stein G. S., Stein J. L., van Wijnen A. J. (1998). Transcriptional control of osteoblast differentiation. *Biochemical Society Transactions*.

[27] Kieswetter K., Schwartz Z., Hummert T. W. (1996). Surface roughness modulates the local production of growth factors and cytokines by osteoblast-like MG-63 cells. *Journal of Biomedical Materials Research*.

[28] Frerich B., Lindemann N., Kurtz-Hoffmann J., Oertel K. (2001). In vitro model of a vascular stroma for the engineering of vascularized tissues. *International Journal of Oral and Maxillofacial Surgery*.

[29] Rausch-Fan X., Qu Z., Wieland M., Matejka M., Schedle A. (2008). Differentiation and cytokine synthesis of human alveolar osteoblasts compared to osteoblast-like cells (MG63) in response to titanium surfaces. *Dental Materials*.

[30] Raines A. L., Olivares-Navarrete R., Wieland M., Cochran D. L., Schwartz Z., Boyan B. D. (2010). Regulation of angiogenesis during osseointegration by titanium surface microstructure and energy. *Biomaterials*.

[31] Lohmann C. H., Bonewald L. F., Sisk M. A. (2000). Maturation state determines the response of osteogenic cells to surface roughness and 1,25-dihydroxyvitamin D3. *Journal of Bone and Mineral Research*.

[32] Bonewald L. F., Kester M. B., Schwartz Z. (1992). Effects of combining transforming growth factor *β* and 1,25-dihydroxyvitamin D_3_ on differentiation of a human osteosarcoma (MG-63). *The Journal of Biological Chemistry*.

[33] Majeska R., Rodan G. A. (1982). The effect of 1,25(OH)2D3 on alkaline phosphatase in osteoblastic osteosarcoma cells. *The Journal of Biological Chemistry*.

[34] Lossdörfer S., Schwartz Z., Wang L. (2004). Microrough implant surface topographies increase osteogenesis by reducing osteoclast formation and activity. *Journal of Biomedical Materials Research Part A*.

[35] Schwartz Z., Olivares-Navarrete R., Wieland M., Cochran D. L., Boyan B. D. (2009). Mechanisms regulating increased production of osteoprotegerin by osteoblasts cultured on microstructured titanium surfaces. *Biomaterials*.

[36] Gurlek A., Pittelkow M. R., Kumar R. (2002). Modulation of growth factor/cytokine synthesis and signaling by 1*α*,25-dihydroxyvitamin D_3_: implications in cell growth and differentiation. *Endocrine Reviews*.

[37] Mundy G. R. (1991). The effects of TGF-beta on bone. *Ciba Foundation Symposium*.

[38] Naves M., Álvarez-Hernández D., Fernández-Martín J. L. (2003). Effect of VDR gene polymorphisms on osteocalcin secretion in calcitriol-stimulated human osteoblasts. *Kidney International, Supplement*.

[39] Lim J. S., Kim K. M., Rhee Y., Lim S.-K. (2012). Gender-dependent skeletal effects of vitamin D deficiency in a younger generation. *Journal of Clinical Endocrinology and Metabolism*.

